# Paths of Cultural Systems

**DOI:** 10.3390/e20010008

**Published:** 2017-12-25

**Authors:** Paul Ballonoff

**Affiliations:** Owner and Operator, Ballonoff Consulting, 9307 Kings Charter Drive, Richmond, VA 23116, USA; Paul@Ballonoff.net; Tel.: +1-703-780-1761

**Keywords:** quantum logic, groups, partially defined algebras, quasigroups, viable cultures

## Abstract

A theory of cultural structures predicts the objects observed by anthropologists. We here define those which use kinship relationships to define systems. A finite structure we call a partially defined quasigroup (or pdq, as stated by Definition 1 below) on a dictionary (called a natural language) allows prediction of certain anthropological descriptions, using homomorphisms of pdqs onto finite groups. A viable history (defined using pdqs) states how an individual in a population following such history may perform culturally allowed associations, which allows a viable history to continue to survive. The vector states on sets of viable histories identify demographic observables on descent sequences. Paths of vector states on sets of viable histories may determine which histories can exist empirically.

## 1. Ethnographic Foundation

The structures described here and their consequences imply much of what may be predicted about empirical cultures. Anthropologists very often draw illustrations of structures using methods discussed here, but based on intuition, thus have little notion of what their commonly used diagrams might predict. While [1,2] defined mathematical means to describe the current and future demographic organization of lineage organizations, with empirical examples, we here specify the demography of kinship-based systems [3,4,5,6,7,8] with some related definitions in our Appendix A; and empirical examples in [9,10,11,12,13]. We follow the inspiration of [14]. In an empirical culture many other relations may also occur; we note some of those in our Part 7, Discussion at the end. 

Our notion of studying viable minimal structures—which are the smallest minimal cultural structures that can “reproduce” the ascribed social relations in one generation—follows from [15]. Our history describes how sustaining those relations allow the culture to reproduce the rules. Cultural rules describing histories may be stated in natural languages, which label the individuals in a descent sequence with a subset called a kinship terminology. Our term viable embodies what anthropologist Radcliff-Brown called “persistent cultural systems” ([10], p. 124). Radcliff-Brown and others often described histories using discrete generations, as do we. Empirical cultures are almost ubiquitously described by many anthropologists using viable histories, typically represented in an ethnography by its minimal structure. The nearly ubiquitous presence in ethnographies of viable histories implies they may be the only observed histories.

**Figure 1:**
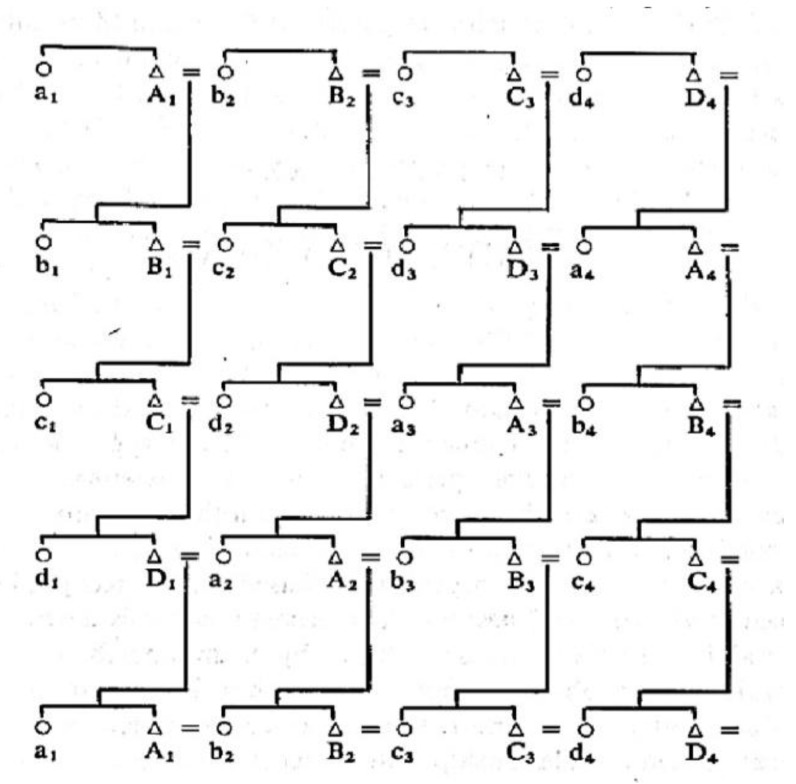
Example of a typical anthropological illustration of a persistent cultural system per Radcliff-Brown, which is also a group per Levi-Strauss and Weil.

An example is Figure 1 (whose source is [9]) which is an actual viable minimal structure of a history (that is, a persistent cultural system). The triangles in the illustration are males, the circles are females, descent moves in the downward direction, the labels are names used in the kinship terminology. The sign “=” means “marriage” between the two individuals attached to it; the “=” on the far right in the illustration shows a marriage to the partner on the far left in each generation. The horizontal line which connects two individuals shows that those two are assigned descendants of the marriage above them. The fifth generation in this illustration is equal to the first (by its labels in vertical descent in the diagram), showing that minimal structure shown here reproduces the labelled culture here in four generations, but reproduces the minimal structure (the graphs without the kinship labels) in each generation. This minimal structure has 4 marriages in each generation hence has structural number *s* = 4. The minimal structure is not intended to illustrate the actual empirical relations of each empirical generation of individuals, instead it shows “how the rule operates”—it describes the minimal representation of the kinship and marriage rules (see Appendix A Definitions A3). The minimal structure describes the “principles” used in the rules of the culture.

Figure 1 is also obviously an example of a group. Claude Levi-Strauss [9] initially used groups to demonstrate histories in an appendix by A. Weil. Ref. [9] mainly discusses groups of orders 2, 4 and 8, though its illustration 1.17 shows a helical structure of order 4. Other studies include orders 3, 6 and others. Ref. [16] also shows a helical structure of order 7; helical minimal structures are also groups and have surjective descent sequences, so our modal demography discussed in [8] and Appendix A Definition A7 also apply to helices. 

## 2. Basic Definitions 

While lineage organizations [1,2] predicted examples of population measures including the “local” village size given the lineage structure, the kinship examples described here use values found in [8] to predict values associated to the structural number of the history, which apply no matter what the empirical size of the total population (so long as it is at or above the minimal size). Our definitions are stated in our Appendix A from previous articles (see also Appendix A) and those below.

**Definition** **1.** Let D be a finite non-empty set (called a dictionary) and let * be a partially defined binary operation on D, such that when x, y ∈ D:

*(1)*  If there exists an a ∈ D such that a*x and a*y are defined and a*x = b and a*y = b then x = y, we call such object (D, *) a partially defined quasigroup, or pdq.
*(2)*  If (D, *) is a pdq and * is fully defined on D, then (D, *) is a (complete) quasigroup.
*(3)*  The pair L = (D, *) is a natural language with dictionary D whenever (D, *) is a pdq.
*(4)*  If L = (D, *) is a natural language, a kinship terminology is a quasigroup subset k ⊆ L.

**Definition** **2.** Let X, Y and Z be non-empty finite sets and let (X, *), (Y, °) and (Z, ▪) be quasigroups with binary relations *, ° and ▪ respectively. Then:

*(1)*  A function f: X → Y is a homomorphism if f for all b, c ∈ X, f(b * c) = f(b) ° f(c).
*(2)*  *If f: X → Z and g: Y → Z are homomorphisms then f and g are isotopic.*

All empirical languages are natural languages [7]. Under Definition 2(2) if Y and Z are isotopic they are also istotopic dictionaries: two possibly different descriptions, thus typically of distinct natural languages, of the “same” objects or illustrations. Our definition of kinship terminologies based on quasigroups follows from [17], which refined the discussion of Weil in [9]. While empirical kinship systems are non-associative [18], a large class is associative and complete, form finite permutations, indeed groups [19,20,21] hence form kinship terminologies as defined here. Groups thus arise in anthropology because a set of all 1-1 mappings of a finite subset set *k* of a quasigroup (a kinship terminology *k*) onto itself forms a group (the symmetric group on *k*). If (X, *), (Y, °) and (Z, ▪) are complete pdqs then (isotopic) homomorphisms classify kinship terminologies by the form of the pdq onto which they are mapped. For example, the isotopic terminologies classified as Dravidian [12,22,23] and others are often discussed, in part because they have interesting group theoretical structures.

**Definition** **3.** *Let H be a non-empty finite set of viable histories. Let G be a non-empty descent sequence using H, let G_t_ ∈ G be a generation of G at t, and let H_t_ ⊆ H be a subset of t. Then then for each α ∈ H_t_, the real numbers 0* ≤ *v_α_(t)* ≤ *1 such that* Σ*_α_v_α_(t) = 1 is the vector state of G_t_*.

Adopting a standard order for listing the histories, we write the vector state at *t* as *v*(*t*) := (*v_α_*(*t*), …, *v_χ_*(*t*)), or when |*H_t_*| = *h*, as *v*(*t*) = (*v*_1_(*t*), …, *v_h_*(*t*)). Let *H *be a finite non-empty set of viable histories, let *α* ∈ *H*, let *G_t_ ∈ G* be a generation of G, and let *v*(*t*) be the vector state of *G_t_* (see also Appendix A Definitions A2–A5). Then:

- From [2,5,6,8] and Appendix A Definition A4 each structural number *s* has a set of values *n_s_* and *p_s_* where *n_s_p_s_* = 2, where *n_s_* is the average family size of a pure system of structural number *s* and *p_s_* is the proportion of reproducing adults of a pure system of structural number *s*. If history *α* has structural number *s*, then each *α* has modal demography (*n_α_*, *p_α_*) = (*n_s_*, *p_s_*) (see Appendix A Definition A7) where *p_s_* = 2/*n_s_*; for *s* ≥ 3 and *s_α_* ≠ *s_χ_* then (*n_α_*, *p_α_*) ≠ (*n_χ_*, *p_χ_*).
- Determination of the (*n_s_*, *p_s_*) values are based on the Stirling Number of the Second Kind (SNSK) see [8,24]. We assume here the (*n_s_*, *p_s_*) pairs determined by [8].
- Since *H* is finite, each non-empty set of viable histories *H* thus has a largest structural number *s_max_* with modal demography (*n_max_*, *p_max_*), and a smallest structural number *s_min_* with modal demography (*n_min_*, *p_min_*). Note that if n_max_ increases then *p_max_* decreases (and as *n_min_* decreases then *p_min_* increases, since given *s*, *n_s_p_s_* = 2, with 0 < *p* ≤ 1. Structural numbers *s* = 2 or 3 have identical modal demography (*n_s_*, *p_s_*) = (2, 1); all others structural numbers have distinct modal demographies see [5,8].
- The modal demography of history α with structural number *s*_α_ is (*n*_α_, *p*_α_) = (*n_s_*, *p_s_*) is a set of values that represent the history α maintaining its modal demography with neither increase nor decrease in total empirical population size; it is prediction of *n*_α_ and *p*_α_ based on the determination that the structural number is *s*, and maintains the structural number *s*.
- *n*(*t*) = Σ*_α_v_α_*(*t*)*n_α_*, *α* ∈ *H*, is the predicted average family size of *G_t_* at *t*, given the vector state at *t* see [8]. Note that this is the average family size of the population at time *t*, given the vector state of each α ∈ *H_t_*. This while the “size” of the minimal structure might be small, the size predicted by *n*(*t*) is the predicted actual size of the total population at *t*, not of the minimal structure; the minimal structure illustration “size” is dependent on the rules, not on the empirical size of the population.
- *p*(*t*) = Σ*_α_v_α_*(*t*)*p_α_*, α ∈ *H*, is the predicted proportion of reproducing adults of *G_t_* at *t* ascribed as married and reproducing, given the vector state of *s_α_* at *t* [8].
- Thus, all of the “demographics” of cultural theory discussed here are predictions on the result of maintaining or changing the vector states of *t*, given the SNSK determined values for each modal demography (*n_α_*, *p_α_*) at time *t*. Thus, [8] defines
  *e^r^*^(*t*)^ = 1/2*n*(*t*)*p(t*) (1)
  where *r*(*t*) ∈ *R* predicts an average rate of change of total population size between two generations of *G*, based on the vector state of structural numbers of the histories *H_t_* ⊆ *H*. [8] showed that *r*(*t*) predicts changes in the probabilities *v*(*t*) imply cultural change is adiabatic.
- Let *H* be a finite non-empty set of viable histories, and *α*, *χ* ∈ *H*. Using *v_α_*(*t*) = 1 − *v_χ_*(*t*), *n_α_* = 2/*p_α_* and *n_χ_* = 2/*p_χ_*, then Equation (1) becomes
  *e*^*r*(*t*)^ = 1 + (*n_αχ_* – 2)*v_α_*(*t*) + (2 – *n_αχ_*)*v_α_*(*t*)^2^ (2)
  where:
  *n_αχ_* := (*n_α_*^2^ + *n_χ_*^2^)/(*n_α_n_χ_*) (3)
  is a constant determined by the values of *n_α_* and *n_χ_*; note *n_αχ_* = *n_χα_*.

## 3. Paths of Descent Sequences

**Definition** **4.** *Let H be a finite set of non-empty viable histories. Let α, χ, etc ∈ H_t_ ⊆ H and let the structural number of α ≠ χ, etc. If for any such set, |H_t_| > 1, v_t_(α) = 1 or 0, then H is not full; otherwise H is full*.

**Definition** **5.** *If H is a finite non-empty set of viable histories, then F ⊆ H is a face of H see [2,4,8]. Let I = [1, 0]. A path from point a to point b in a set X is a function f: I → X with f(0) = a and f(1) = b, in which case a is called the initial point of the path and b is called the terminal point of the path. Given a path, in case a = b then such path is a closed path. If [x, y] ∈ I and f(x) = a and f(y) = b then f[x, y] is called an interval and a sub-path of I. A reverse path from point a to point b in X is a function f: I → X with f(1) = a and f(0) = b. Given a path (or reverse path) from t_0_ to t_1_, if t_1_ ≥ t_k_ ≥ t_0_ we say that t_k_ is in the path*.

**Lemma** **1.** *Let H_t_ be a finite non-empty set of full viable histories and α, χ ∈ H_t_. Then:*

*1.*  *r(t) ≥ 0, and r(t) = 0 if all structural numbers have the same modal demography or if all have structural numbers 2 or 3.*
*2.*  *If α and χ are distinct structural numbers and at least one has structural number >3, then r(t) > 0 and r(t) has a maximum at v(t) = (0.5, 0.5).*
*3.*  Given any finite non-empty set H of two or more viable histories, there is a unique maximum r(t), given by (ii).

**Proof** **1.** Assume first the modal demographies of histories *α* and *χ* are equal (which occurs if *s_α_* = *s_χ_* or if *s_α_*, *s_χ_* < 4). Then *n_α_* = *n_χ_*. Then *n_αχ_* = 2, so the sum in Equation (2) is *e*^*r*(*t*)^ = 1, or *r*(*t*) = 0. Assume now s_α_ ≠ s_χ_ and at least one structural number is >3, so the modal demographies of α and χ are distinct, and we do not have *v_α_*(*t*) = 0 or 1. Then in Equation (2) (*n_αχ_* − 2) = −(2 − *n_αχ_*), but *v_α_*(*t*) > 0, so then *v_α_*(*t*) > *v_α_*(*t*)^2^. Thus, (*n_αχ_* − 2)*v_α_*(*t*) > (2 − n_αχ_)v_α_(t)^2^, so Equation (2) states *r*(*t*) > 0. ☐

**Proof** **2.** To show *r(t)* has a maximum when *v*(*t*) = (0.5, 0.5), we use the first two terms of a Taylor expansion from (1) to find *e*^*r*(*t*)^ = 1 + *r*(*t*). Differentiating twice gives *a*^2^ exp[*r*(*t*)]/*δy^2^* = 2 − 2*n_αχ_* where *y* = *v*(*t*). When s_α_ ≠ s_χ_ and at least one of *s_α_*, *s_χ_* is > 3, *n_αχ_* > 2, then exp[*r*(*t*)] is concave down; so, *r*(*t*) is also concave down. ☐

**Proof** **3.** There is a finite number of two-history pairs in *H*. Since *n_s_* increases as *s* increases, then Lemma 1 part 1 shows that the largest value of *r*(*t*) will be set by that two-history subset *α*, *χ* of *H* having the largest difference between their structural numbers, hence the largest *n_αχ_* in Equation (3). Combining the *n_s_* of the largest *s* with any other combination of *n_s_* values will result in a smaller *n_αχ_* hence smaller *e*^*r*(*t*)^, from Equation (3). ☐

**Figure 2:**
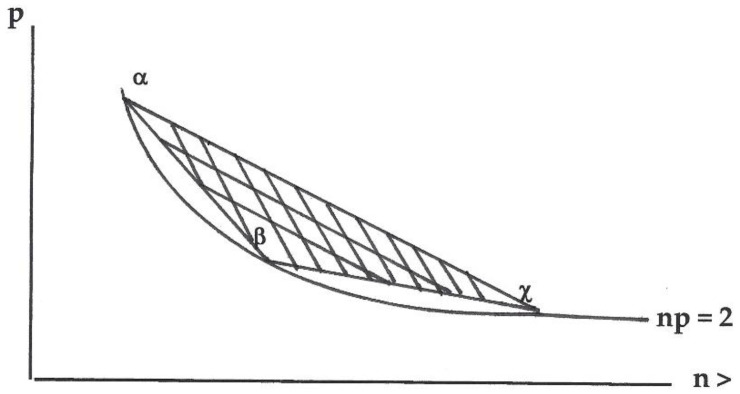
Illustration of the curve *np* = 2 showing three histories and connections among those three histories at or above that curve.

**Observation** **1.** Assume a finite non-empty set of viable histories *H* acting on a finite non-empty descent sequence *G*. Let *α*, *β*, *χ* ∈ *H_t_* ⊆ *H* act on generation *Gt* ∈ *G*. See Figure 2:

The curved bottom-line in Figure 2 is the locus wherever *np* = 2, so includes the modal demography of each history in *H*; that is, the modal demography for each of *α*, *β* and *χ* each appear on the line *np* = 2, since *n_s_p_s_* = 2. If for any *α*, *v_α_*(*t*) = 1, then *n*(*t*) = *n_α_*, *p*(*t*) = *p_α_*, so *e^r^*^(*t*)^ = ½*n*(*t*)*p*(*t*) = ½*n_α_p_α_* = ½2 = 1 so *r*(*t*) = 0; this occurs if all histories in *H_t_* have the same structural number (or all have structural numbers 2 and 3). When that does not occur we have a set of two or more histories in *H_t_* each with 0 < *v_α_*(*t*) < 1 and thus *r*(*t*) > 0; see also Lemma 1. When *H* is full, assume *α*, *β* and *χ* have distinct structural numbers, at least two of *α*, *β*, *χ* have structural numbers >3, and α is has the lowest structural number of those three histories. Then the modal demography of *α*, *β*, *χ* have (*n_α_*, *b_α_*) ≠ (*n_β_*, *b_β_*) ≠ (*n_χ_*, *b_χ_*), and the computation of *r*(*t*) appears in the values in the triangle area of Figure 2. However, if *H_t_* is not full then events in the triangle area might not occur. Even if paths allow *α* with *β*, *α* with *χ* and *β* with *χ* (thus the boundaries of the triangle area), values of *r*(*t*) within the triangle only occur if all three histories are allowed by *H*, which may be prohibited by not-full *H*. We call such area an un-accessed region. Thus, we study change using both full and non-full sets of histories. 

## 4. Pictures of States on Descent Sequences

**Definition** **6.** Let H be a finite non-empty set of histories and let (n_α_, p_α_) be the modal demography for history α ∈ H. Let G be a finite non-empty descent sequence using H, and let G_t_ ∈ G be the generation at time t. Let H_t_ be a face of H specified at t. Let S_t_ := { s_α_|α ∈ H_i_} be the set of structural numbers S_t_ ⊆ S of histories H_t_ available at t. Let **A**_t_ := {(n_α_, p_α_)|s_α_ ∈ S_i_, α ∈ H_i_, (n_α_, p_α_) = (n_s_, p_s_)} be the set of modal demographies **A**_t_ of the histories in H_t_; and let v(t) be the vector state of G_t_. List the histories in H in a defined order from α to χ. Then for all α ∈ H_t_ and all (n_α_, p_α_) ∈ **A**_t_, let:

*1.*  *(n_t_|:= (n_α_, …, n_χ_) be a row vector;*
*2.*  |p_t_) := (p_α_,…, …, p_χ_) be a column vector;
*3.*  *for all α, χ* *∈ H, arranging the sum of the inner product (n_t_|p_t_) as a square matrix then for all α, χ* *∈ H, H(t): = [n_α_p_χ_] is a demographic picture (analogous to a Heisenberg picture in physics) at t;*
*4.*  *the square matrix we get by arranging the products of v(t)v(t)^T^ as V(t) := [v_αχ_(t)] is a probability picture (analogous to a Schroedinger picture in physics) of the vector state of a descent sequence at t;*
*5.*  *for ε ≥ 0 let V(Δ(t)) := V(t + ε) − V(t) = [v_αχ_(t + ε) − v_αχ_(t)] := [Δ_αχ_(t)]. (Notice that −1 ≤ Δ_αχ_(t) ≤ 1)*.
  *We note [25] for our analogy of terminology*.

Then for all α, *χ* ∈ *H* we can rewrite Equation (1) as:

*e*^*r*(*t*)^ = ½ (*n|V*(*t*)|*p*) (4)

using a probability picture which focuses on the vector states; and

*e*^*r*(*t*)^ = ½*v*(*t*)*H*(*t*)*v*(*t*)^T^ (5)

using a demographic picture which focuses on demographic properties of the histories.

## 5. Comments on Demographic Pictures

Given a finite non-empty set of full viable histories *H*, observing a face *H_t_* ⊆ *H* at *t* produces a list of the available *H_t_* ⊆ *H* and thus creates a list of possible modal demographies (*n_α_*, *p_α_*) ∈ ***A****_t_* for all *α* ∈ *H_t_*. Let |*H_t_*| = *h*. List the *h* histories in a fixed order from *α* to *χ*, with row vector (*n_t_*| = (*n_α_*, …, *n_χ_*) and column vector |*p_t_*) = (*p_α_*, …, *p_χ_*)^T^. So for histories *α*, …, *χ* ∈ *H_t_*, we can write the demographic picture for |*H_t_*| = *h* at *t* as (*n_t_*|*p_t_*) = *H*(*t*) where:

(6) $$H\left(t\right)=\left(\begin{array}{ccc} n_{1}p_{1} & \ldots & n_{1}p_{h}\\ \vdots & \ddots & \vdots \\ n_{h}p_{1} & \cdots & n_{h}p_{h} \end{array}\right)=\left(\begin{array}{ccc} 2 & \ldots & n_{1}p_{h}\\ \vdots & \ddots & \vdots \\ n_{h}p_{1} & \cdots & 2 \end{array}\right)$$

Each diagonal entry = 2 because a diagonal entry *n_α_p_α_* is determined by the modal demography (*n_s_*, *p_s_*) for each history, and *n_s_p_s_* = 2. Thus, using ½*H*(*t*) we can restate Equation (5):

(7) $$\begin{array}{ll} e^{r(t)} & =½v\left(t\right)H\left(t\right)v{\left(t\right)}^{T}=½v\left(t\right)\left(\begin{array}{ccc} 2 & \ldots & n_{1}p_{h}\\ \vdots & \ddots & \vdots \\ n_{h}p_{1} & \cdots & 2 \end{array}\right)v{\left(t\right)}^{T}\\ & =v\left(t\right)\left(\begin{array}{ccc} 1 & \ldots & ½n_{1}p_{h}\\ \vdots & \ddots & \vdots \\ ½n_{h}p_{1} & \cdots & 1 \end{array}\right)v{\left(t\right)}^{T} \end{array}$$

Because the two-history case has some useful properties, we present much of our discussion on the two history version, which becomes:

(8) $$e^{r(t)}=v\left(t\right)\left(\begin{array}{ccc} 1 & & ½n_{1}p_{2}\\ ½n_{2}p_{1} & & 1 \end{array}\right)v{\left(t\right)}^{T},\;\mathrm{where}\;\;½H\left(t\right)=\left(\begin{array}{ccc} 1 & & ½n_{1}p_{2}\\ ½n_{2}p_{1} & & 1 \end{array}\right).$$

**Lemma** **2.** *Let H be a finite non-empty set of full viable histories. Let G be a non-trivial descent sequence using H, let H_t_ ⊆ H be the face of H observed at t, and let G(t) ∈ G be the generation at t with vector state v(t). Then r(t) = 0 only if ½H(t) = [1] at all entries*.

**Proof of Lemma** **2.** Assume the premises. Equations (4)–(7) simply rearrange terms in ½Σ*_i_*Σ*_j_v_i_*(*t*)*v_j_*(*t*)*n_i_p_j_*. From the definition of modal demography, *p_i_* = 2/*n_i_* and *p_j_* = 2/*n_j_*. The values on the diagonal of ½*H*(*t*) are for each history *α*, ½*n_α_p_α_* = 1. We thus examine the off-diagonal products *n_α_p_χ_* and *n_χ_p_α_*. Then *n_i_p_j_* = 2*n_i_*/*n_j_* and *n_j_p_i_* = 2*n_j_*/*n_i_*. Thus, 2n_i_/*n_j_* = 2*n_j_*/*n_i_* occurs only if *n_i_* = *n_j_*, in which case *n_i_p_j_* = *n_j_p_i_* = 2. This occurs only if all histories i and j have the same structural number or both have *s* = 2 or 3, and thus ½*H*(*t*) = [1] in all entries. Otherwise stated, in this case the value from Equation (3) is *n_αχ_* = 2. ☐

Implications of Lemma 2: knowing the modal demography of histories in *H* we can compute a proposed population growth rate *r*(*t*).

1. The result *n_α_* = *n_χ_* occurs if structural numbers *s_α_*, *s_χ_* are <4 or whenever *s_α_* = *s_χ_*; so r(*t*) = 0. Otherwise, then Lemma 2 implies Lemma 1, which says that *e^r^*^(*t*)^ ≠ 1, and thus *r*(*t*) > 0. This occurs since *n_α_p_χ_* does not equal *n_χ_p_α_*; thus from Lemma 1 and [8] the off-diagonal elements of ½*H*(*t*) implies adiabatic change in *r*(*t*).
2. In discussions in physics, when *n_α_p_χ_* ≠ *n_χ_p_α_* some claim that the resulting *r*(*t*) is “not commutative”. In physics, the “non-commutative” result actually means switching which experiment is taken, then comparing their results; in physics when changing the order of the products it also means changing the experiment; but this comparison of the two results also creates an equation that looks like our Equations (1), (2), (7) or (8). However, in physics reversing the experiment causes different measurements, which causes the physical uncertainty between the two results. In contrast, the seemingly “non-commuting” values in culture theory exist because the equation for computing *r*(*t*) requires computing both “directions” of the modal demography of histories in *H* (similar to comparing both directions of the physics model), and if any two (or more) of those have histories of distinct structural numbers (at least one >3), so that one or more *n_αχ_* > 2 (see Equation (3)), then *n_χ_p_α_* ≠ *n_α_p_χ_*. Culture theory thus predicts adiabatic demographic change, not uncertainty, from a mechanism similar to that which causes uncertainty in physics.

## 6. Comments on Probability Pictures

**Lemma** **3.** *A probability picture V(t) is symmetric, Σ_i_Σ_j_v_i_(t)v_j_(t) = 1 and Σ_i_Σ_j_(Δ_ij_(t)) = 0*.

**Proof of Lemma** **3.** In Equation (4) *V*(*t*) is symmetric since each pair *v_i_*(*t*)*v_j_*(*t*) = *v_j_*(*t*)*v_i_*(*t*). Since Σ*_α_v_α_*(*t*) = 1 then *v*(*t*)*v*(*t*)^T^ = Σ*_i_*Σ_j_*v_i_*(*t*)*v_j_*(*t*) = 1. At *t* + *ε* ≥ *t* (*ε* < *t* − (*t* − 1)) then Σ*_α_v_α_*(t + *ε*) = 1: so Σ*_i_*Σ*_j_v_i_*(*t* + *ε*)*v_j_*(*t* + *ε*) = 1; so Σ*_i_*Σ*_j_*(v_ij_(*t* + *ε*) − *v_ij_*(t)) = Σ*_i_*Σ*_j_*(Δ*_ij_*(*t*)) = 1 − 1 = 0. ☐

Since we discuss paths of histories, a frequency-domain representation of vector states is useful.

**Definition** **7.** *Let r_1_, r_2_, r_3_ be real numbers such that r_1_^2^ + r_2_^2^ + r_3_^2^ = 1. Let R be a set of 2 by 2 matrices with complex entries that forms a ring with respect to matrix addition and multiplication. Let **R** ⊆ R be a set of hermitian idempotent matrices of R; and let R ∈ **R** be such that R = ½[r_ij_] where r_11_ = 1 + r_3_, r_22_ = 1 − r_3_, r_21_ = r_1_ + ir_2_, r_12_ = r_1_ − ir_2_. That is*:

$$R=½\left[r_{ij}\right]=½\left(\begin{array}{cc} 1+r_{3}\; & r_{1}-ir_{2}\\ r_{1}+ir_{2} & 1-r_{3} \end{array}\right)$$

Following ([26], p. 30) we define matrices

$$1=\left(\begin{array}{cc} 1 & 0\\ 0 & 1 \end{array}\right),\Sigma_{1}=\left(\begin{array}{cc} 0 & 1\\ 1 & 0 \end{array}\right),\Sigma_{2}=\left(\begin{array}{cc} 0 & -i\\ +i & 0 \end{array}\right),\Sigma_{3}=\left(\begin{array}{cc} 1 & 0\\ 0 & -1 \end{array}\right)$$

and let *z*_0_, *z*_1_, *z*_2_, *z*_3_, be complex numbers such that *R* = *z*_0_1 + *z*_1_Σ_1_ + *z*_2_Σ_2_ + *z*_3_Σ_3_ where:

*z*_0_ = ½(*r*_11_ + *r*_22_), *z*_1_ = ½(*r*_21_ + *r*_12_), *z*_2_ = *i*½(*r*_21_ − *r*_12_), *z*_3_ = ½(*r*_11_ − *r*_22_).

The four matrices 1, Σ_1_, Σ_2_, and Σ_3_ are the standard Pauli spin matrices, where for *R* then *z*_0_ = 1, *z*_1_ = *r*_1_, *z*_2_ = *r*_2_ and *z*_3_ = *r*_3_. Note that −1 ≤ r_1_, r_2_, r_3_ ≤ 1. From ([27], p. 104) *R* is a set of non-trivial 2 by 2 version of *R*; a ring of such forms an orthomodular poset and indeed an atomic orthomodular lattice with the covering property, that is in 1-1 correspondence with the set of closed subspaces of a two-dimensional complex Hilbert space.

**Definition** **8.** *Let H be a finite non-empty set of viable histories, let G be a non-trivial viable descent sequence using histories H_t_ ∈ H, let G_t_ ∈ G be a generation of G using a face H_t_ ∈ H at t, and let v(t) be the vector state of G_t_*.

1. Let _2_*H_t_* = {*α, χ*} *⊆ H_t_* be a two-history subset of *H_t_*. Let *R*(*t*) = ½[*r_ij_*(*t*)] be a projection, let *r*_1_(*t*), *r*_2_(*t*), and *r*_3_(*t*) be real numbers such that r_1_(*t*)^2^_+_ *r*_2_(*t*)^2^ + *r*_3_(*t*)^2^ = 1, such that 0 ≤ *r*_1_(*t*) < 1, 0 ≤ *r*_2_(*t*) < 1, and such that *v_α_*(*t*) = ½*r*_1_(*t*) = ½(1 + *r*_3_(*t*)). Then *R*(*t*) is the *status* of *G_t_*.
2. A *unit circle C* is meant a set of points (*x*, *y*) in the plane R^2^ which satisfy the equation *x*^2^ + *y*^2^ = 1.

**Theorem** **1.** Assume the premises of Definition 8. Let H be a finite non-empty set of viable histories having structural numbers s < 152 (see [8] for use of this limit). Let G be a descent sequence using H. Let R(t) be the status of H_t_, and let v(t) be the vector state of H_t_. Let _2_H_t_ = {α, χ} ⊆ H_t_ be a non-empty subset of H_t_. Let t_2_ > t_1_ > t_0_ define a path of v_α_(t) from t = t_0_ to t = t_2_ such that v_α_(t_0_) = 1 changes monotonically to v_α_(t_1_) = 0 and then monotonically back to v_α_(t_2_) = 1. That is, let r_3_ move from r_3_(t_0_) = 1 to r_3_(t_1_) = − 1 and then back to r_3_(t_2_) = 1. Let O(t) = (n(t), p(t), r(t)). Then:

*(1)*  *trR(t) = 1;*
*(2)*  *v_χ_(t) = ½(1 − r_3_(t));*
*(3)*  *the vector state v(t) of _2_H_t_ is given by the main diagonal of R(t);*
*(4)*  *r(t) is a maximum when r_3_ = 0*.

**Theorem** **2.** *Let r_1_(t) = 0. Then: (i) R(t) has ΣΣ_ij_r_ij_(t) = 1; and (ii) the sum Σ∫r(t)dv(t) = 0 when summed over all paths (all variants of paths) of for pairs _2_H_t_*.

**Theorem** **3.** *Let r_2_(t) = 0. Then Σ∫r(t)dv(t) = 0 when summed over all paths (all variants of paths) of all pairs _2_H_t_*.

**Proof of Theorem** **1.** Assume the premises of Theorem 1. In a two history system, _2_*H_t_* = {*α*, *χ*} ⊆ *H_t_* is the vector state *v*(*t*) = (*v_α_*(*t*), *v_χ_*(*t*)) where *v_χ_*(*t*) = 1 − *v_α_*(*t*). *R*(t) is a status and since in a status *v_α_*(*t*) = ½*r*_11_ = ½(1 + *r*_3_), and since *v_α_*(*t*) + *v_χ_*(*t*) = 1 in a two-history state, then *v_χ_*(*t*) = ½*r*_22_ = ½(1 − *r*_3_). In addition, also then *v_α_*(*t*) + *v_χ_*(*t*) = ½(1 + *r*_3_) + ½(1 − *r*_3_) = 1 = *trR*(*t*), which establishes Theorems 1, 2, and 3. Establishing 4: We find *r*(*t*) is a maximum when r_3_ = 0, given Theorem 1(1) and 1(3), and Lemma 1(2), so when *r*_3_ = 0 then *v*(*t*) = (0.5, 0.5).

Let *r*_1_(*t*) = 0 so

$$R(t)\;=\;½[r_{ii}]\;=\;½\left(\begin{array}{cc} 1+r_{3} & -ir_{2}\\ ir_{2} & 1-r_{3} \end{array}\right)$$

and thus ½Σ*_i_*Σ*_j_r_ij_*(*t*) = ½2 = 1 which establishes 1(1). 

Let *H_t_* = {*α*, *χ*}. At time *t*, *H_t_* picks a set of modal demographies ***A****_t_* = {(n_α_, *p_α_*), (*n_χ_*, *p_χ_*)} and *v*(*t*) acts as a linear operator on ***A****_t_*; so we get

*v*(*t*)***A****_t_* = Σ*_α_v*(*t*)(*n_α_*, *p_α_*) = (*n*(*t*), *p*(*t*)) for all α ∈ *H_t_*.

From Lemma 2, *O*(*t*) = (*n*(*t*), *p*(*t*), *e*(*t*)) are the predicted results at *t*; when *s_α_* ≠ *s_χ_* then (*n_α_*, *p_α_*) ≠ (*n_χ_*, *p_χ_*). *R*(*t*) is an idempotent Hermitian matrix per Definition 7, and under the premises has *r*_1_ = 0. Then *r*_2_^2^ + *r*_3_^2^ = 1. We have a two history system with vector state *v*(*t*) = (*v_α_*(*t*), *v_χ_*(*t*)) where *v_χ_*(*t*) = 1 − *v_α_*(*t*), and where *v_α_*(*t*) = ½r_11_ = ½(1 + *r*_3_(*t*)). We let *t*_2_ > *t*_1_ > *t*_0_ define a path from *t* = *t*_0_ to *t* = *t*_2_ such that *v_α_*(*t*_0_) = 1 changes monotonically to *v_α_*(*t*_1_) = 0 and then monotonically again to *v_α_*(*t*_2_) = 1, which occurs as *r*_3_(*t*) moves monotonically from *r*_3_(*t*) = 1 to *r*_3_(*t*) = −1 and then back to *r*_3_(*t*) = 1. At each *t*, given *r*_3_(*t*), we compute *r*_2_(*t*)^2^ = 1 − *r*_3_(*t*)^2^. Then *r*_2_(*t*)^2^ + *r*_3_(*t*)^2^ = 1 traces a unit circle C. Theorems 2 and 3 then follow from Green’s theorem. □

**Observation** **2.** *Let G be a population, G_t_ ∈ G with the sub-populations G_t_ using the set of histories using face H_t_ ∈ H, and let v(t) be the vector state of G_t_. Assume history α ∈ H_t_, α ∈ H_t+1_, and v_α_(t) = v_α_(t + 1) = 1. Then from t to t + 1, v(t) forms a loop. That is, the minimal descent sequence of any viable pure system α also forms a loop, indicated also since the minimal structure of α is a group. So any pure system is a loop. Diagrams like Figure 1 could occur when v(t) is not simply a pure system. Describing probability pictures by complex Hilbert spaces (Definition 7) can assist predicting demographic pictures, using pure systems as the basis of computing n(t), p(t) hence r(t)*.

## 7. Discussion 

Following the examples of [1,2] we here study systems in which the cultural organization is based on kinship descriptions using natural languages. In both cases, our theory makes predictions on population measures on the observed outcome of the kinship systems at stated times. Our Observation 1 and Lemma 1 predict what is found empirically: either single history systems and specific (*n_s_*, *p_s_*) pairs by the structural number of the identified history; or systems undergoing change in their culture. In that case the (*n_s_*, *p_s_*) pairs are changing and we can predict that rate of change yielding both the *n(t*) and *p*(*t*) for the given *t*, and the value of the adiabatic growth rate *r*(*t*) at *t*. An example of this prediction of rate of change in western Europe for about 1000 years from about AD 1000 to 1950 is given in [1]. The time period of that study was about 1000 years of human history in a defined area. 

Thus, study of the homotopy groups resulting from Definitions 5, 7 and 8 may thus tell us a lot about the possible paths of the empirical demography of cultures. Definitions 7 and 8 assume no physical model, but we can use their math to study the changes in vector states on histories on the predicted *n(t*), *p(t*) and *r(t*) of the society per generation. The methods of [28,29] and many other current works such as [30] in social sciences use complex Hilbert spaces to describe models of how “cognition” works, using much shorter time periods, and to otherwise interpret how societies of individuals can describe and change the world around them. Hilbert space probability models per [31], which is a foundation paper for [13], are quite close to the Pauli model used here to describe changes in cultural systems; they differ from ours in their application. In particular, [31], Postulate 4 does not apply here since the applications are distinct. However, the probabilities of [30,31] are averages of probabilities on a population, not predictions of individual probabilities. There may be thus be many ways to discuss evolution of cultural systems using complex Hilbert spaces that have simply not yet been tried. 

In this paper, in [17], and in both [30,31] the mathematical foundation starts with representation of the basic objects as languages; ours are natural languages. Kinship systems are derived from non-associative algebras [20,32] which in natural languages may allow groups to occur. Cultural systems with different dictionaries but similar groups are studied as isotopic kinship terminologies for example [8,11], which is a separate topic mathematically and empirically from study of languages [9,10,11,12,13]. Ref. [33] says “… kinship organizations are based on terminologies, which have their own distinctive logical structure centered on a “self” or I position. Language does not have a structure of this kind …”. So while kinship terminologies occur as part of natural languages, kinship analysis is not the same as the study of the language.

Our study also helps identify what cannot be predicted by this method. For example, sociologists and anthropologists use relationship studies to describe how individuals are “related”; the minimal structure defined here based on assignments made based on the “principles” used to arrange or avoid marriages, given the natural language and the history; they do not define which specific individuals are in fact assigned to each relation. In contrast, in genetic inbreeding experiments Sewall Wright [34] at diagrams 7.1(a), 7.12, 7.16 and others used the minimal structure of kinship relations for illustrating inbreeding arrangements; but in those situations, the individuals are not “assigned”—they are the actual kin of the identified sources. 

The ability to derive population measures from the language-based statement of rules is something new to science, and should be explored. Many other things also affect population change, and are not explored here. 

## Appendix A. Mathematical Background from Previous Papers

For convenience of use here, this appendix adopts background previously presented mainly in [8].

**Definition** **A1.** *General mathematical usages. Let L be a finite non-empty set. A partial order ≤ is a binary relation on L such that for a, b, d ∈ L, a ≤ a; a ≤ b and b ≤ a implies a = b; and a ≤ b and b ≤ d implies a ≤ d. Then (L, ≤) is a partially ordered set or poset. A lattice is a poset on which is defined two binary relations join ∪ and meet ∩ such that for a, b ∈ L then a ∪ b ∈ L and a ∩ b ∈ L. A lattice (L, ≤) is bounded if L contains special elements **0** and **1** such that for b ∈ L, **0** ≤ b ≤ **1**, which we denote as (L, ≤, **0**, **1**). An involution is a unary relation ’ on L such that for b ∈ L, then b’ ∈ L, b = b” and for b, d ⊆ L if b ≤ d then d’ ≤ b’. An object (L, ≤, ’, **0**, **1**) is a bounded involution poset. If L is a bounded involution poset an orthogonality relation* ⊥ *on L is a binary relation such that for b, d ∈ L, b* ⊥ *d if b ≤ d’*.

**Definition** **A2.** Properties of populations and descent sequences:

**Definition** **A2.1.** *Let G be a finite non-empty set called a population whose members d ∈ G are called individuals. Let D (descent), B (sibling of) and M (marriage) be binary relations on G, satisfying these four axioms: (1) D is anti-symmetric and transitive; (2) M is symmetric; (3) if bDc and there exists no d ∈ G, d ≠ b, c for which bDd and dDc, then we write cPb, and require bBc iff for b, c, d ∈ G, dPb and dPc; (4)|bM | ≤ 2*.

**Definition** **A2.2.** *Let G(t) = {G_t_|G_t_ ⊆ G, t ∈ T, G_i_ ∩ G_j_ = ∅ for i ≠ j} is a family of subsets of G, indeed a partition of G, where t ∈ T is a set of consecutive non-negative integers starting with 0; such G(t) is a descent sequence of G*.

**Definition** **A2.3.** *G_t_ ⊆ G is called the t^th^ generation of G, in case, for all G_t_ ∈ G, each cell bB occurs in only one generation, each subset bM occurs in only one generation, and for t > 0 when G_t_ ∈ G, b ∈ G_t_, and cPb, then c ∈ G_t−*1*_ (that is, G_t_ contains all of and only the immediate descendants of individuals in G_t−*1*_). Let |G_t_| = δ_t_*.

**Definition** **A2.4.** *Let G_t_ be a descent sequence of G. Let B_t_ := {bB|b ∈ G_t_, G_t_ ∈ G, t > 0} be a partition of G_t_. in which each bB is a sibship; and let M_t−1_ := {bM|b ∈ G_t_, G_t_ ∈ G, t ≥ 0} be a set of disjoint subsets of G^t−*1*^ in which each bM is a marriage. Let |B_t_| := β_t_, and |M_t_| := μ_t_*.

We allow that only at *t* = 0 may there be individuals in a generation that did not arise by descent from a previous generation of *G*. For *b ∈ G*, any set *bM* is assumed to be reproducing. Other than *t* = 0, members of *G_t_* arise from (assignment of offspring to) marriages in *G_t−_*_1_, thus *β_t_* = *μ_t_*_−1_.

**Definition** **A3.** *Properties of configurations*:

**Definition** **A3.1.** *Let G(t) be a descent sequence, G_t_ ∈ G be a generation of G, and let a, b, c, ..., k ∈ G_t_, a ≠ b ≠ c ≠ … ≠ k be individuals in G_t_. Then a regular structure is a closed cycle aBb, bMc, cBd, …, kMa of a finite number of alternating B and M relations, in which each a ∈ G_t_ occurs exactly twice in such a list, being exactly once on the left of a B followed immediately by once on the right of an M, or once on the right of a B preceded immediately by once on the left of an M, and in such cycle each |bB| = |bM| = 2. If there are j instances of M in such a cycle, then the regular structure is of type Mj*.

**Definition** **A3.2.** *Given a finite positive integer k, we assume a set of unit basis vectors e_i_, 1 ≤ i ≤ n, such that in e_i_ the ith position = 1 and all others = 0; and write c = (m_1_, m_2_, …, m_k_). If G_t_ ∈ G is a generation and m_i_ is the number of regular structures of type Mi in G_t_, then such a c is called a configuration*.

**Definition** **A3.3.** *Let c = (m_1_, m_2_, …, m_k_) be a configuration. Then μ_c_ := ∑_i_(m_i_i) is the number of marriages of c*.

**Definition** **A3.4.** *Let C := (c|c is a configuration} be the set of configurations. Let M ∈ R^+^. Let C_M_ := {c|μ_c_ ≤ M} be a set of configurations of order M*.

For example, a configuration with a single M2 structure would be written (0, 1, 0, 0, …). Note that the null configuration 0 := (0, 0, …, 0) *∈ C_M_*. If *c ∈ C_M_*, *c* ≠ 0, then such a *c* is non-null, and if *B* ⊆ *C_M_* contains at least one non-null configuration, then such a *B* is non-null.

Configurations are often used in ethnography when describing kinship systems. A set *bM* identifies a reproducing marriage in a configuration which has offspring in the succeeding generation. In a configuration we ignore all non-reproducing individuals who may exist “empirically” in *G_t_*. So while |*bB*| ≥ 1 in general, |*bB*| = 2 is required in a configuration. If we let |*G_t_*| = *δ_t_*, the number of individuals in a configuration on *G_t_* is *γ_t_* = 2*μ_t_*; so *δ_t_* ≥ *γ_t_* and *δ_t_* ≥ 2*μ_t_*; so *n_t_* = *δ_t_*/*β_t_* = *δ_t_*/*μ_t_*_−1_. Since individuals in *G_t_* arise only from reproducing marriages among individuals of *G_t-1_*, then *β_t_* = *μ_t_*_−1_. Thus:

**Definition** **A4.** *If G_t_ is a generation of G, then n_t_ := δ_t_/β_t_ is the average family size of G_t_*.

**Definition** **A5.** *Properties of histories*:

**Definition** **A5.1.** *A history α is a binary relation on P(C_M_), that is, (c,d) ∈ α ⊆ P(C_M_) × P(C_M_); such an α induces a function of the power set of P(C_M_) which we also call α, defined, for B ∈ P(C_M_) _,_ by (c,d) ∈ α(B) = {d ∈ C_M_|c α d for some c ∈ C_M_}. We let H_M_ := {α|α is a history defined on C_M_} be the set of all histories on C_M_*.

**Definition** **A5.2.** *A configuration c ∈ C_M_ is viable under α if there exists an integer k > 0 such that c ∈ α^k^(c)*.

**Definition** **A5.3.** *A history α ∈ H_M_ is viable if there is at least one c ∈ C_M_, c ≠ 0, such that c is viable under α. Let V(α) := {c|c ∈ C_M_ and c is viable under α} be the set of all viable configurations under α. If 0 is the only configuration viable under a history α, then such an α is called trivial; otherwise α is called non-trivial*.

**Definition** **A5.4.** *If α is a viable history then s_α_ := min({μ_c_|c ∈ V(α)\{0}) is the structural number of α}, where S := {s_α_|α ∈ H_M_ } is a set of structural numbers of viable histories in H_M_. If c_α_ ∈ V(α) such that μ_c_ = s_α_ then c_α_ is a minimal structure of α. If α is a viable history, let Min_α_ := {c ∈ V(α)|μ_c_ = s_α_} be the set of minimal structures*.

**Definition** **A5.5.** *Let c, d ∈ C_M_, and let η_c_ ∈ H_M_ be a viable history such that η_c_(c) = {c} and η_c_(d) for c ≠ d is not defined; then η_c_ is a pure system. Let H_p_ := {η_c_|c ∈ C_M_} be the set of pure systems on C_M_. If α is a history, G(t) is a descent sequence of α, then c ∈ Min _α_ and c = c_t_ for all G_t_ ∈ G then G is a pure system of α*.

With the usual set union ∪ and intersection ∩ then the power set (*P*(*C_M_*), ∪, ∩) is a Boolean algebra, and using ≤ as a partial order by set inclusion, (*P*(*C_M_*), ≤, 0, *C_M_*) is a poset, indeed a bounded involution poset. A history is thus a natural language describing “how *α* works to create a history”. Here, a history is a rule; specifically a marriage rule. The structural number *s_α_* of a viable history *α* is simply the value of *μ* of a smallest configuration which is viable under *α*; so also *s_α_* > 0, indeed |*V*(*α*)| ≥ 2. Notice that *Min*_α_ ⊆ *V*(*α*) and |*Min*_α_| ≥ 1.

**Definition** **A6.** *Let G_t_ ⊆ G be a non-empty generation of G at time t, let |G_t_| = n, and let G_t_ be partitioned into 1 ≤ k ≤ n subsets. Then:*

(i) *We call a pair (n, k) an assignment.*
(ii) *A set of assignments is a selection denoted by **A** with subsets A ⊆ **A**. To specify more detail of the membership of a set A we may also use subscripts or a square bracket notation [n, k] with subscripts as required.*
(iii) [n, k] := {(n, k)|given a positive integer n, (n, k) where 1 ≤ k ≤ n}.
(iv) [n, k]_j_ := {(n, k)|given a finite positive integer j, (n, k) where 1 ≤ n ≤ j and for each n, 1 ≤ k ≤ n}.
(v) [n, k]_j,i_ := {(n, k)|given finite integers i, j where i ≥ j, (n, k) for 1 ≤ n ≤ j and 1 ≤ k ≤ i}.
(vi) P_j_ := P([n, k]_j_) denotes the set of subsets of [n, k]_j_.
(vii) *If (n_1_, k_1_), (n_2_, k_2_) are assignments such that n_1_ ≠ n_2_ or k_1_ ≠ k_2_, then (n_1_, k_1_) and (n_2_, k_2_) are distinct assignments.*
(viii) *If **A** is a set of assignments, is a unary relation on A ⊆ **A** such that A’ := **A**\A.*
  *If (n, k) is an assignment, then*
(ix) n := n/k is the average family size of (n, k).
(x) p := 2/n is the reproductive ratio of (n, k).

*Since np = 2, then 0 < p ≤ 1*.

**Definition** **A7.** *Let (n, k) be an assignment*.

(i) *S(n, k) is a Stirling Number of the Second Kind, where*
  (9) $$S\left(n,\;k\right)=\frac{1}{k!}\overset{k}{\underset{j=0}{\sum }}{\left(-1\right)}^{k-j}\left(\begin{array}{c} k\\ j \end{array}\right)j^{n}$$
  *is the number of ways to partition a set of n distinct elements into k nonempty subsets. S(n, k) computes the number of ways to achieve an assignment (n, k) for n individuals in a generation partitioned into k = β_t_ = μ_t−1_ ≥ s_α_ non-empty subsets [J] which we call families. Then:*
(ii) S[n, k] := {S(n, k)|for given n, S(n, k), k = 1, …, n} is called a distribution.
(iii) *Given a distribution S[n, k], then [n, k] := {(n, k)| for given n, k = 1, …, n} is the underlying selection of S[n, k].*

*Since S(n, k) = 1 when n = k then (uniquely) for n = 2 the distribution S[2, k] is bimodal, with modes at k = 1, 2. Therefore for n > 2:*

(iv) n^↑^ := {j | given n}.
(v) n^↑^_s_ := {n^↑^ | j = s, for a given s > 0}.
(vi) *S[n, n^↑^_s_] := {S(n, n^↑^)|given s, n^↑^ ∈ n^↑^_s_}.*
(vii) *N_s_ := n|S(n, n^↑^) = max(S[n, n^↑^_s_]).*
(viii) ***A****_[s]_ := ∪ [n, k] for n such that n^↑^ ∈ n^↑^_s_ and for each such n, 1 ≤ k ≤ n, called the minimal collection of s.*
(ix) ***A****_M_ := ∪ **A**_[s]_, given a positive integer M, for structural numbers 1 < s ≤ M.*
(x) m_s_ := (N_s_, s) is the modal assignment for s.
(xi) ***A****_s_ := {m_s_ | s ∈ **S**} is the set of modal assignments for s ∈ S.*
(xii) n_s_ := N_s_/s is the modal average family size for s.
(xiii) p_s_ := 2/n_s_ is the modal reproductive ratio for s.
(xiv) *(n_s_, p_s_) is the modal demography of s*.
